# Retraction Note to: Diffusion-weighted MR imaging of locally advanced breast carcinoma: the optimal time window of predicting the early response to neoadjuvant chemotherapy

**DOI:** 10.1186/s40644-021-00434-2

**Published:** 2021-11-25

**Authors:** Li Yuan, Jian-Jun Li, Chang-Qing Li, Cheng-Gong Yan, Ze-Long Cheng, Yuan-Kui Wu, Peng Hao, Bing-Quan Lin, Yi-Kai Xu

**Affiliations:** 1 Department of Medical Imaging Center, Nanfang Hospital, Southern Medical University, #1838 Guangzhou Avenue North, Guangzhou City, 510,515 Guangdong Province China; 2 Department of Radiology, Hainan General Hospital, Haikou, 570,311 Hainan Province China

**Retraction Note to: Cancer Imaging 18, 38 (2018).**

**https://doi.org/10.1186/s40644-018-0173-5**

The Editors-in-Chief have retracted this article. After publication concerns were raised that there were discrepancies in the reporting of the number of NACT cycles the patients underwent. The authors were given the opportunity to provide an explanation but have not responded to any correspondence regarding this matter. The Editors-in-Chief therefore no longer have confidence in the results presented.

Li Yuan, Jian-Jun Li, Chang-Qing Li, Cheng-Gong Yan, Ze-Long Cheng, Yuan-Kui Wu, Peng Hao, Bing-Quan Lin and Yi-Kai Xu have not responded to any correspondence from the editor/publisher about this retraction.

